# Supplementary material for: Conversion of Glycerol to Value Added Products in a Semi-Continuous Batch Reactor Using Noble Metals Supported on ZSM-11 Zeolite
Source: Nanomaterials (Basel). 2021 Feb 17;11(2):510. doi: 10.3390/nano11020510 (PMC7922169; doi:10.3390/nano11020510)
Supplement: Supplementary file 1 [file nanomaterials-11-00510-s001.pdf]

Supporting Information

Conversion of Glycerol to Value Added Products in a Semi-Continuous Batch Reactor Using Noble Metals Supported on ZSM-11 Zeolite

Eliana Diguilio [1], M. Soledad Renzini [1], Liliana B. Pierella [1,*] and Marcelo E. Domine [2,*]

[1] Centro de Investigación y Tecnología Química (CITeQ), UE CONICET–Universidad Tecnológica Nacional, Facultad Regional Córdoba, Maestro Lopez esq Cruz Roja Argentina, Ciudad Universitaria, (5016) Córdoba, Argentina; ediguilio@frc.utn.edu.ar (E.D.); mrenzini@frc.utn.edu.ar (M.S.R.)

[2] Instituto de Tecnología Química (UPV-CSIC), Universitat Politècnica de València, Consejo Superior de Investigaciones Científicas, Avda. de los Naranjos s/n, 46022 Valencia, Spain

* Correspondence: lpierella@frc.utn.edu.ar (L.B.P.); mdomine@itq.upv.es (M.E.D.); Tel.: +54-351-469-0585 (L.B.P.); +34-963-879-696 (M.E.D.)

Table of Contents

S1. TEM images, particle size distribution (a) and XEDS spectra (b) of the bimetallic catalyst: Au-Pt- ZSM-11.

S2. TEM images, particle size distribution (a) and XEDS spectra (b) of the bimetallic catalyst: Au-Pd- ZSM-11.

S3. Temperature programmed reduction (TPR analysis) of monometallic catalysts a) Au-ZSM-11, b) Pt-ZSM-11, c) Pd-ZSM-11.

S4. GLY conversion and selectivity (mol%) towards LA, GA and DHA over different Metal/ZSM-11.

S1. TEM images, particle size distribution (**a**) and XEDS spectra (**b**) of the bimetallic catalyst Au-Pt- ZSM-11.

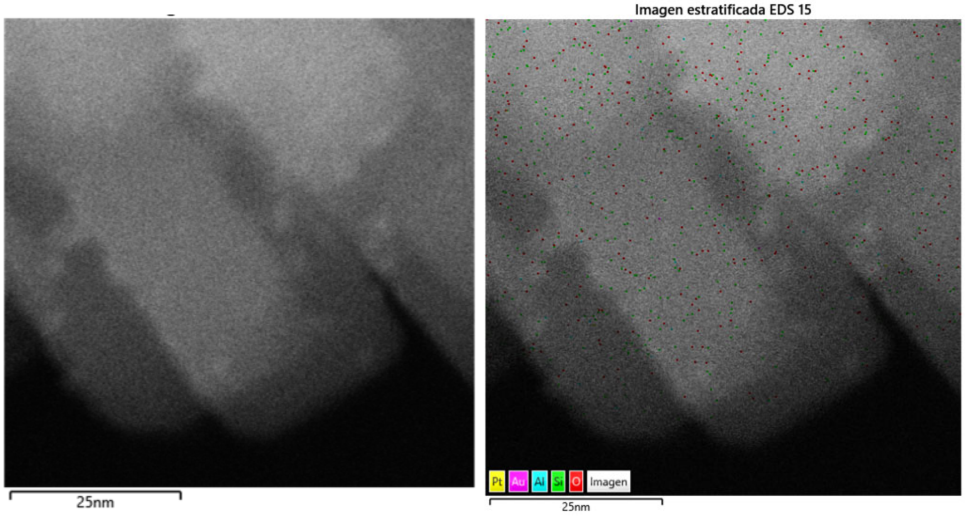

a)

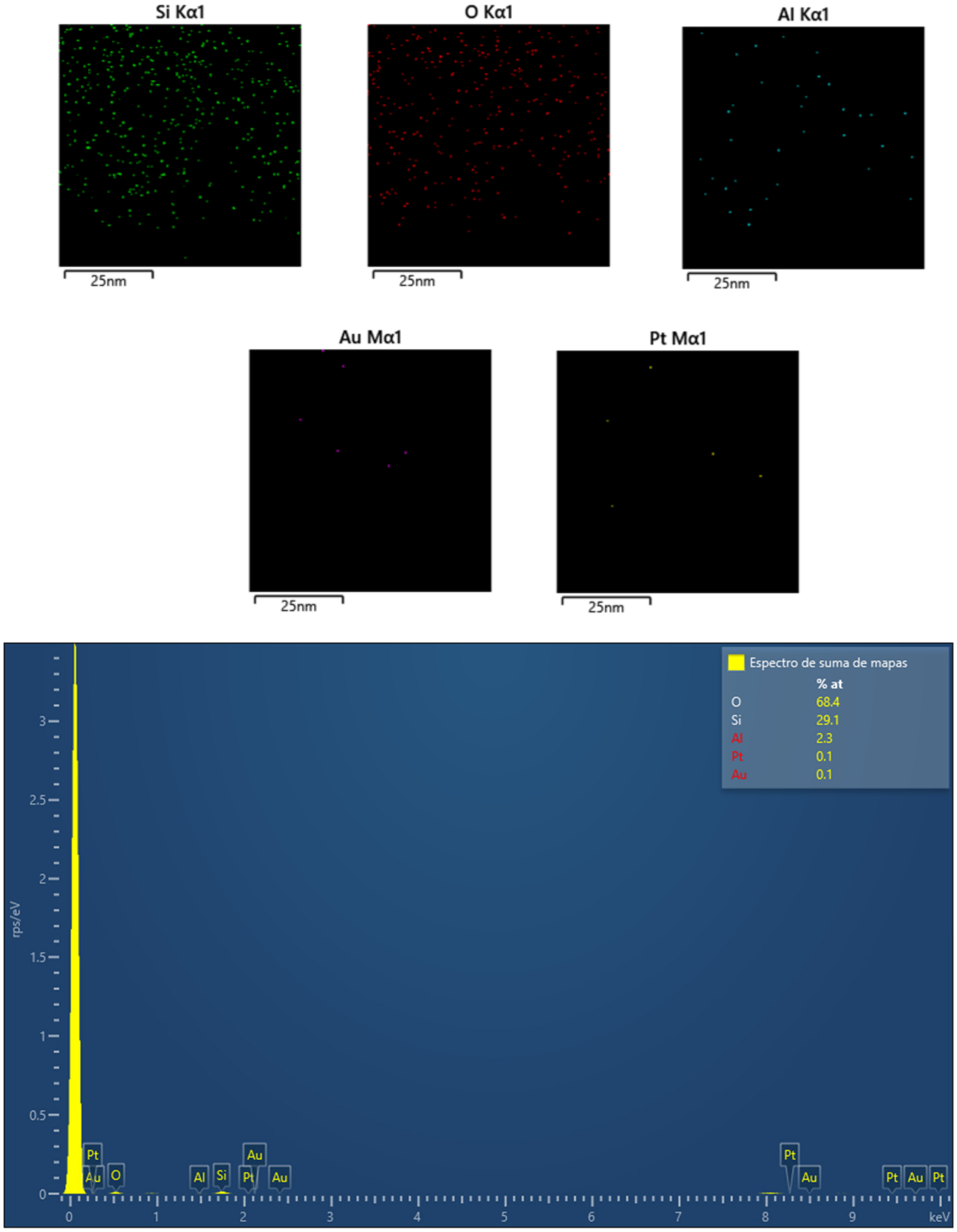

b)

S2. TEM images, particle size distribution (**a**) and XEDS spectra (**b**) of the bimetallic catalyst Au-Pd- ZSM-11.

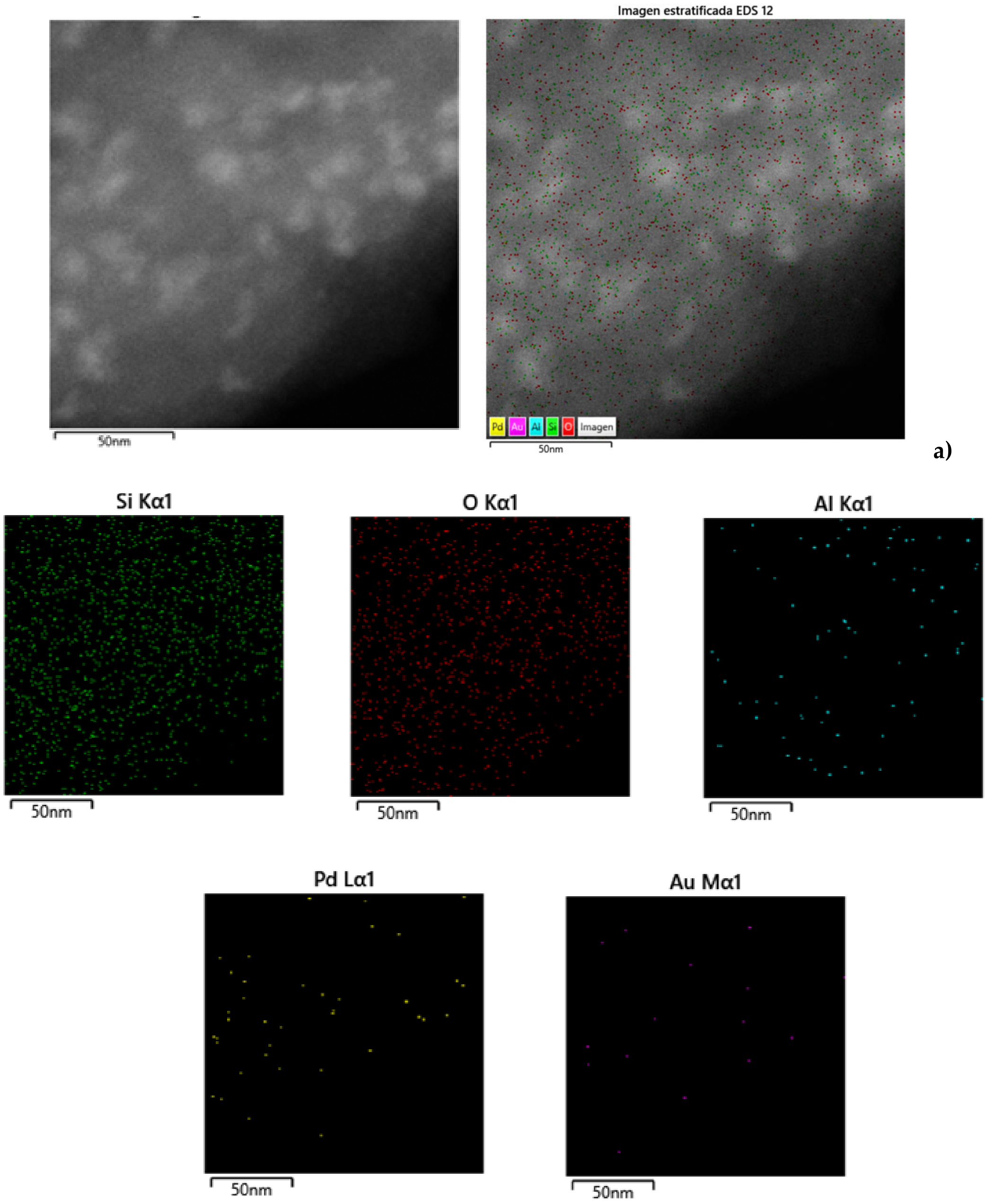

Imagen estratificada EDS 12
Pd
Au
Al
Si
O
Imagen
50nm
a)
Si Kα1
O Kα1
Al Kα1
Pd Lα1
Au Mα1

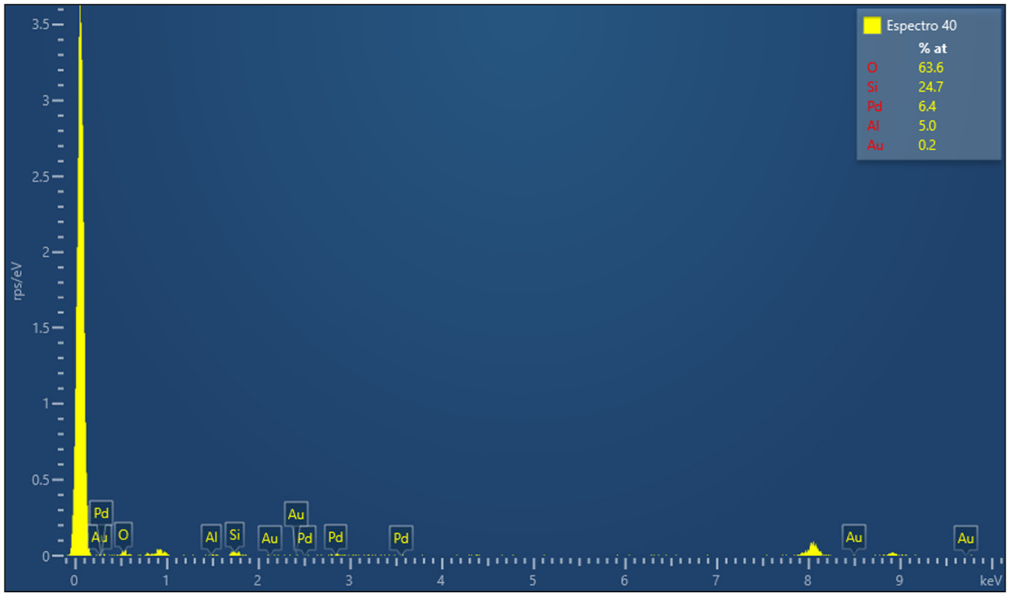

b)

S3. Temperature programmed reduction (TPR analysis) of monometallic catalysts (**a**) Au-ZSM-11, (**b**) Pt-ZSM-11, (**c**) Pd-ZSM-11.

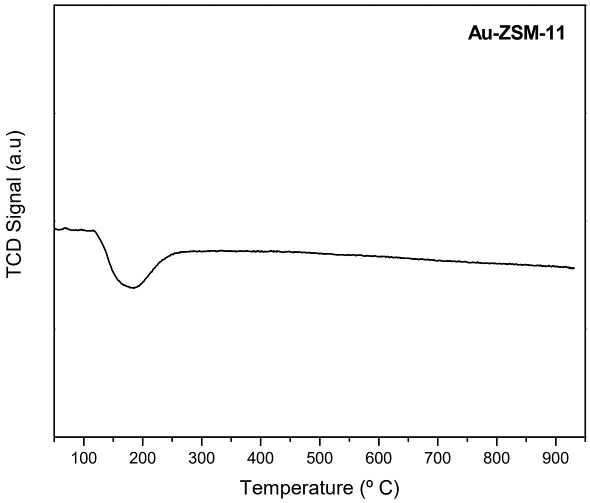

a)

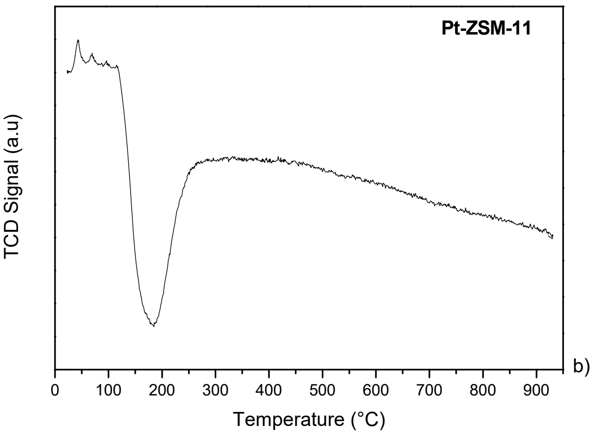

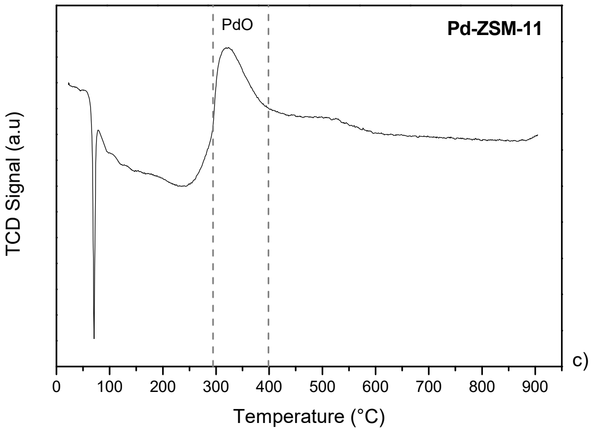

S4. GLY conversion and selectivity (mol.%) towards LA, GA and DHA over different Metal/ZSM-11.

At 60 min of reaction

Catalyst	Reaction Time (min)	GLY Conversion (mol%)	Selectivity (mol%) LA	GA	DHA
Pt -ZSM-11 [a]	60	51.9	16.5	-	-
Pd-ZSM-11 [a]	60	36.9	-	71.9	-
Au-ZSM-11 [a]	60	46.8	-	41.4	-
Au-Pt-ZSM-11 [a]	60	16.8	8.9	20.5	-
Au-Pd-ZSM-11 [a]	60	16.0	7.3	19.5	-
Cu-ZSM-11 [b]	60	27.1	68.0	-	19.3
Cu-ZSM-11 (T) [b]	60	24.1	51.4	-	3.1
Cr-ZSM-11 [b]	60	13.7	-	-	11.4
Cr-ZSM-11 (T) [b]	60	23.8	10.2	-	18.1

At 120 min of reaction

Catalyst	Reaction Time (min)	GLY Conversion (mol%)	Selectivity (mol%) LA	GA	DHA
Pt -ZSM-11 [a]	120	56.6	25.4	-	-
Pd-ZSM-11 [a]	120	32.8	-	63.9	-
Au-ZSM-11 [a]	120	48.4	-	48.5	-
Au-Pt-ZSM-11 [a]	120	21.0	36.9	25.1	-
Au-Pd-ZSM-11 [a]	120	16.0	12.2	22.5	-
Cu-ZSM-11 [b]	120	42.5	49.0	-	25.2
Cu-ZSM-11 (T) [b]	120	31.5	41.5	-	5.0
Cr-ZSM-11 [b]	120	40.6	-	-	28.3
Cr-ZSM-11 (T) [b]	120	42.5	5.3	-	27.5

a- Reaction conditions: 35 mL sol. GLY (0.25 M), NaOH/GLY = 2 (mol/mol), GLY/metal = 400 (g/g), at 70 °C and atmospheric pressure.

b- Reaction conditions: 30 mL sol. GLY (0.5 M), H_2O_2/GLY = 2 (mol/mol), GLY/metal = 230 (g/g), at 60 ºC and atmospheric pressure.

S5. GLY conversion and selectivity (mol%) towards majority products over different noble metals supports.

Catalyst	Reaction Condition	X GLY (%)	S GA (%)	S DHA (%)	Ref.
Au/C	Sol. GLY (0.3 M, 30 mL), T= 100 °C, 11 bar O_2, NaOH/GLY = 2 (mol/mol), t= 0.5 h	6.8	67	-	[1]
Pd/C		29	83	-	[1]
Pt/C		16	70	-	[1]
Pt/AC	Sol. GLY (0.3M, 50 mL), T= 60 °C, 150 mL/min O_2, t = 6 h	50	47.4	-	[2]
Pt/CNTs	Sol. GLY (0.1 M, 50 g), T= 60 °C, 150 mL/min O_2, 600 rpm	22	19.5	10.4	[3]
Pd-Au/C	Sol. GLY (5%w/w, 20 g), T= 80 °C, 0.3 Mpa O_2, t = 4 h	6.1	-	47.8	[4]
Au-Pt/MgO	Sol. GLY (0.3 M, 15 mL), T = 80 °C, 300 KPa O_2, t = 4 h, GLY/metal = 1000 (mol/mol)	45	40	-	[5]
Au-Pt/SiO_2	Sol. GLY (0.3 M), T= 80 °C, 3 atm O_2 GLY/metal = 500 mol/mol	30	61	18	[6]

X: conversion of GLY ; S: Selectivity

References

1. Zope, B.N.; Hibbitts, D.D.; Neurock, M.; Davis, R.J. During Selective Oxidation Catalysis *Science* **2010**, *330*, 74–79. doi: 10.1126/science.1195055.
2. Liang, D.; Gao, J.; Wang, J.; Chen, P.; Hou, Z.; Zheng, X. Selective oxidation of glycerol in a base-free aqueous solution over different sized Pt catalysts. *Catal. Commun.* **2009**, *10*, 1586–1590, doi:10.1016/j.catcom.2009.04.023.
3. Gao, J.; Liang, D.; Chen, P.; Hou, Z.; Zheng, X. Oxidation of glycerol with oxygen in a base-free aqueous solution over Pt/AC and Pt/MWNTs catalysts. *Catal. Letters* **2009**, *130*, 185–191, doi:10.1007/s10562-009-9849-6.
4. Hirasawa, S.; Watanabe, H.; Kizuka, T.; Nakagawa, Y.; Tomishige, K. Performance, structure and mechanism of Pd-Ag alloy catalyst for selective oxidation of glycerol to dihydroxyacetone. *J. Catal.* **2013**, *300*, 205–216, doi:10.1016/j.jcat.2013.01.014.
5. Xu, C.; Du, Y.; Li, C.; Yang, J.; Yang, G. Insight into effect of acid/base nature of supports on selectivity of glycerol oxidation over supported Au-Pt bimetallic catalysts. *Appl. Catal. B Environ.* **2015**, *164*, 334–343, doi:10.1016/j.apcatb.2014.09.048.
6. Villa, A.; Campisi, S.; Mohammed, K.M.H.; Dimitratos, N.; Vindigni, F.; Manzoli, M.; Jones, W.; Bowker, M.; Hutchings, G.J.; Prati, L. Tailoring the selectivity of glycerol oxidation by tuning the acid-base properties of Au catalysts. *Catal. Sci. Technol.* **2015**, *5*, 1126–1132, doi:10.1039/c4cy01246a.
